# Seasonal distribution and population dynamics of limnic microalgae and their association with physico-chemical parameters of river Noyyal through multivariate statistical analysis

**DOI:** 10.1038/s41598-019-51542-w

**Published:** 2019-10-21

**Authors:** Pandian Suresh Kumar, Jibu Thomas

**Affiliations:** Algae Biomass Research Laboratory, Department of Biotechnology, Karunya Institute of Technology and Sciences, Coimbatore, 641114 Tamil Nadu India

**Keywords:** Environmental biotechnology, Environmental impact

## Abstract

The present investigation embarks on understanding the relationship between microalgal species assemblages and their associated physico-chemical parameter dynamics of the catchment region of river Noyyal. Totally, 142 microalgae cultures belonging to 10 different families were isolated from five different sites during four seasons and relative percentage distribution showed that Scenedesmaceae (36.6%) and site S1 (26.4%) with predominant microalgae population. Diversity indices revealed that microalgae communities were characterized by high Hʹ index, lower Simpson dominance, and Margalef index value with indefinite patterns of annual variations. Results showed that variation in the physico-chemical parameters in each sampling site has its impact on the microalgae population during each season. Multivariate statistical analysis viz., Karl Pearson’s correlation coefficient, principal component analysis, and canonical correspondence analysis were applied on microalgae species data, to evaluate the seasonal relationship between microalgae and physico-chemical parameters. The findings of our study concluded that the physico- chemical parameters influenced the dominant taxa of microalgae Chlorellaceae, Scenedesmaceae and Chlorococcaceae in river Noyyal and gives a base data for the seasonal and dynamic relationship between environmental parameters and microalgae population.

## Introduction

Algae are the prominent living entity of aquatic ecosystems and play a vital role in nutrient cycling as they are key players in the sequestration of inorganic nutrients and thereby converting them into organic forms^1,2^. Exploring the prevailing native organisms of varied microhabitats and their massive study collection offers a scope of discovery for a benefitting worldwide biotechnological innovation and application. Native species are generally adapted to the existing environmental conditions and thus they are evolutionarily reformed to bioresource production platforms. In addition, indigenous algae form a vital platform for additional strain development, optimization and facilitating the development of a regional base for algae cultivation.

The physico – chemical parameters in the water bodies highly influences the microalgae population and its diversity^3^ and considered to be major factors that control the population dynamics of microalgae^4^. Alterations in these parameters will have a significant impact on the species availability in water bodies and thereby indicating the quality of water. Also, seasonal variations in these parameters influence their distribution, periodicity and abundance of microalgal population and its diversity^5^. Microalgae are sensitive to these slight modifications in the environment and in such conditions, opportunistic species start multiplying by dominating other species^6,7^ and thereby reducing the actual microalgal species diversity in that habitat^8^.

Diversity indices are the stochastic mathematical functions which explain the number of species in a biological community^9^. In the present study, Shannon–Wiener, Simpson’s dominance, Margalef richness, and evenness have been studied to explain the population status and to understand their community structure. A set of microalgal communities and its associated environmental parameters in a habitat usually yield a huge amount of robust data that is difficult to interpret. Multivariate statistical techniques, such as principal component analysis (PCA), Correlation matrix and canonical correspondence analysis (CCA) can interpret complex data matrices by permitting the identification of potential factors for improved microalgae assemblages, water quality, and other environmental systems^10^. In the present study, PCA is employed to examine the variations between the parameters as variables and to classify them into measurable components. CCA is employed to disclose the relationships between the microalgae assemblages and their stereoscopic environment.

River Noyyal is a venous pulse river in Western Tamil Nadu in South India and a tributary of river Cauvery. It originates from Vellingiri hills in the Western Ghats, Tamil Nadu and flows through many villages and cities finally merges into river Cauvery at Noyyal village (so-called the river name). The other rivers which make up the Noyyal river are Periyar, Sadiyar, and Kanchimanadhi. Periyar river flows out of Siruvani hills and KovaiKutralam, a landmark Waterfalls. Waters of Siruvani from catchment region of river Noyyal has socio-economic importance to the livelihood but received less scientific attention as it is a forest protected area and very few information is available on the characteristics of Siruvani water^11^. So, it is included in the present study area. There are scarce reports on the assessment of water quality based on physico-chemical parameters of the Noyyal river but a report on freshwater microalgae community is absolutely deserted. Our preliminary study indicated a rich diversity among the microalgae of this region and the lack of substantial evidence of this information gains significance in this study. This paper serves as the premium report on the microalgae abundance, distribution and community structure from the catchment region of river Noyyal. The study also emphasizes the influence of seasonal variation on microalgae abundance and delivers the checklist of microalgae species occurrence during major seasons of southern Western Ghats. Hence, the present endeavour was therefore aimed to develop a fresh water algae database of Siruvani water from catchment region of river Noyyal, Western ghats (South India) with the following objectives: (1) to enumerate the diversity, distribution pattern and seasonal variation of microalgae population in different freshwater sites, (2) to study the physico-chemical properties of the freshwater collected in different seasons, (3) to investigate the relationship between the microalgae assemblages and their environment.

## Results

### Seasonal variation in physico-chemical parameters

The diversity of microalgae distribution is mostly influenced by seasonal variation at freshwater (Sadivayal check post S1, Perumalkovilpathi S2, Irutupallam S3) and stagnant water (Perumalkovilpathi S4, Irutupallam S5) sample collection sites of river Noyyal which reflected in varied physico-chemical parameters of the water. In the present study, the pH of the water samples was found to be in the range of 6.16 to 9.54 (Table 1). The highest pH was recorded at site S2 during summer (9.54 ± 0.01) and the lowest pH at site S4 during winter (6.16 ± 0.005). The total alkalinity of freshwaters was found to be in the range 16.66 to 54.66 mg/L during all the seasons with the maximum value recorded in post-monsoon at site S4. The high alkalinity in the site S1 and S2 was due to the presence of carbonate rocks at these sites and the maximum alkalinity at S4 and S2 may be due to increasing in bicarbonate content in the water and high photosynthetic rate which influenced the availability of microalgae in the water sample. The hardness was recorded in the range of 28 ± 4 to 766.6 ± 6.11 mg/L. The turbidity of water fluctuated from 0.5 ± 0.2 to 23.3 ± 0.3 nephelometric turbidity units (NTU). Highest and lowest turbidity was recorded at site S3 during post-monsoon and winter season respectively. Nitrate N was recorded in the range 0.001 to 2.0 mg/L with maximum nitrate N at site S5 during the post-monsoon season. Ammonia N was found in the range 0.003 to 0.839 mg/L with maximum and minimum ammonia N content recorded at S4 during winter and summer season respectively (Table 1). Phosphate was recorded in the range 8.34 ± 0.005 to 854.34 ± 0.289 mg/L and was found to be present only in winter and monsoon season. Our findings also observed that phosphate content in stagnant water at site S4 and S5 was higher than freshwater and was observed maximum at site S4 during winter (854.34 ± 0.289 mg/L). The chloride was found in the range of 4 mg/L to 253 mg/L and exhibited marked seasonal variation being maximum during summer and minimum during the winter season.

**Table 1:** Physico-chemical parameters of Siruvani water from catchment region of river Noyyal collected during a different season. Mean ± standard deviation values were given. NC- not collected.

Parameter	Collection site	Winter	Summer	Monsoon	Post-monsoon
**pH**	S1	6.63 ± 0.01	8.52 ± 0.026	8.16 ± 0.025	8.0 ± 0.08
	S2	NC	9.54 ± 0.011	NC	8.60 ± 0.03
	S3	7.48 ± 0.01	NC	8.06 ± 0.00	8.23 ± 0.07
	S4	6.16 ± 0.005	9.33 ± 0.005	9.11 ± 0.02	8.4 ± 0.05
	S5	7.46 ± 0.005	7.96 ± 0.005	7.66 ± 0.05	7.86 ± 0.01
**Turbidity (NTU)**	S1	15.94 ± 1.79	1.9 ± 0.6	2 ± 0.0	2.66 ± 1.15
	S2	NC	25.7 ± 0.3	NC	2.0 ± 0.0
	S3	0.5 ± 0.2	NC	3.33 ± 0.57	16.33 ± 3.05
	S4	6.5 ± 0.81	1.23 ± 0.05	2.33 ± 0.57	4.66 ± 0.57
	S5	13.06 ± 0.2	23.3 ± 0.3	1.0 ± 0.0	3.0 ± 0.0
**Total Alkalinity (ppm)**	S1	0	46 ± 6.0	31.33 ± 2.3	34.66 ± 3.05
	S2	NC	31.33 ± 9.45	NC	46 ± 0.00
	S3	16.66 ± 3.05	NC	32.66 ± 2.3	32 ± 3.46
	S4	0	34 ± 6.0	34.66 ± 1.15	54.66 ± 1.15
	S5	20.46 ± 1.2	24.66 ± 6.42	42 ± 7.2	35.33 ± 6.11
**Hardness (ppm)**	S1	577.33 ± 26.63	73.33 ± 23.09	68 ± 10.58	28 ± 4.0
	S2	NC	104 ± 0.00	NC	36.0 ± 4.0
	S3	766.66 ± 6.11	NC	46.66 ± 9.23	33.33 ± 6.11
	S4	473.33 ± 22.03	120 ± 13.83	534.66 ± 16.16	44.0 ± 4.0
	S5	200 ± 6.92	536 ± 21.16	401 ± 34.94	445.33 ± 34.94
**Ammonia (ppm)**	S1	0.038 ± 0.001	0.044 ± 0.011	0.027 ± 0.002	0.041 ± 0.003
	S2	NC	0.033 ± 0.055	NC	0.055 ± 0.0
	S3	0.054 ± 0.00	NC	0.035 ± 0.003	0.038 ± 0.001
	S4	0.0839 ± 0.001	0.003 ± 0.001	0	0
	S5	0.015 ± 0.00	0.004 ± 0.001	0	0
**Nitrite(ppm)**	S1	0.0173 ± 0.0	0.163 ± 0.016	0.044 ± 0.002	0.185 ± 0.004
	S2	NC	0.096 ± 0.0	NC	0.556 ± 0.029
	S3	0.001 ± 0.0	NC	0.017 ± 0.002	0.182 ± 0.007
	S4	0.019 ± 0.0	0.181 ± 0.009	0.019 ± 0.0	1.54 ± 0.012
	S5	0.001 ± 0.0	0.007 ± 0.001	0.03 ± 0.0	2 ± 0.048
**Phosphate (ppm)**	S1	8.33 ± 0.005	0	52.08 ± 0.04	0
	S2	NC	0	NC	0
	S3	75.16 ± 0.208	NC	68.63 ± 0.126	0
	S4	854.34 ± 0.289	0	141.66 ± 0.03	0
	S5	58.35 ± 0.026	0	54.2 ± 0.05	0
**Chloride (ppm)**	S1	66 ± 1.15	27 ± 3.6	10 ± 2.64	6 ± 0.0
	S2	NC	18 ± 5.5	NC	15.33 ± 6.11
	S3	18.66 ± 1.15	NC	19 ± 5	9.33 ± 3.05
	S4	12 ± 0.0	29.66 ± 11.59	167.33 ± 2.88	11.33 ± 3.05
	S5	4 ± 0.0	253.33 ± 9.81	12 ± 5	352 ± 2

### Meteorological variations

Our study highlighted the diversity of microalgae-based on their distinct morphological traits and was also concerned about ecology and physiology of the species. Hence, we examined the meteorological responses to varying environmental parameters as an attempt to correlate the responses with the abundance of cultures in the regions. Since the study area received bulk rainfall during the southwest monsoon season, rainfall was considered as an important phenomenon as it brought about changes in the physical and chemical characteristics of the water. The mean annual rainfall per day for the year 2014 was recorded as 2.914 mm. In the present study, rainfall per day was recorded maximum during the post-monsoon season with 10.51 mm and lowest was recorded during summer season (0.15 mm). The atmospheric temperature was recorded minimum (23.46 °C) during winter and maximum (28.97 °C) in summer. Tropical wet areas usually have warmer temperature >28 °C as it gets more direct sunlight with humidity. In the present study, summer season recorded a maximum air temperature owing to the clear sky with higher solar radiation. During the monsoon and post-monsoon season, less solar radiation was observed due to a cloudy sky and more rainfall which vastly reduced the air temperature (Table 2).

**Table 2:** Mean month-wise Meteorological data in catchment region of River Noyyal during the study period.

2014	Rain Day (mm)	Solar Radiation (W/m^2^)	Wind Day Avg (Deg)	Wind Max (Km/hr)	Air Temp Avg (°C)	RH Avg (%)	RH Min Day (%)	RH Max Day (%)
January	0.1	0.178	139.95	5.28	23.46	64.79	37.29	86.30
February	2.125	0.198	139.95	5.581	24.39	62.75	33.98	86.95
March	0.096	57.47	139.95	5.67	26.38	55.02	28.23	82.01
April	0.15	224.22	178.69	7.701	28.97	59.63	30.52	81.03
May	6.40	207.92	217.89	9.85	28.08	66.23	44.28	85.48
June	2.17	198.95	248.73	13.49	27.41	65.50	49.94	78.447
July	4.5	137.53	245.86	13.20	25.64	70.30	54.91	85.64
August	2	166.09	234.86	10.79	25.44	73.05	56.19	87.80
September	4.65	196.40	226.64	10.56	26.03	70.35	50.13	85.62
October	10.51	153.20	182.03	5.60	25.04	81.89	57.03	95.04
November	0.45	131.83	165.06	4.479	24.15	77.82	51.79	93.94
December	1.919	123.89	166.39	4.35	23.50	78.49	51.83	93.73
Mean	2.914	133.15	190.50	8.04	25.71	68.82	45.51	86.83

### Microalgae isolate enumeration and pure culture maintenance

Microalgae were isolated from freshwater (S1, S2, S3) and stagnant water (S4, S5) during different seasons. There is always intense sunlight so algae growth was found to be abundant in these regions. In general, different microalgae prefer different growth media for their growth. So in our study, seven different growth medium were attempted to isolate all the possible number of microalgae cultures present in the water and the cultures with similar morphology found in more than one media were neglected to avoid sibling strains. In total, 142 microalgae cultures (AC 1–142) were isolated from all the seasons and collection sites; and the number of microalgae cultures isolated during each season were found to be 30 (winter), 42 (summer), 28 (monsoon) and 42 (post-monsoon). Among the 142 cultures, 23 cultures were found to be unique based on their morphological characteristics like color, size, shape, etc., and were maintained as Karunya Algae Culture Collection (KACC) 1 to 23. The pure cultures were monitored for every 24 hrs under the light microscope and maintained in the culture room under 27 to 37 µmol m^−2^ s^−1^with the light-dark ratio of 16:8 hours at room temperature. Morphological features based on cell shape, size and color revealed that cultures belong to the family of Chlorellaceae, Asteromonadaceae, Scenedesmaceae, Selenastraceae, Chlorococcaceae, Brachysiraceae, Bacillariaceae, Sphaeropleaceae, Oscillatoriaceae and Prasiolales incertae sedis.

### Microalgae diversity indices

**Figure 1:**
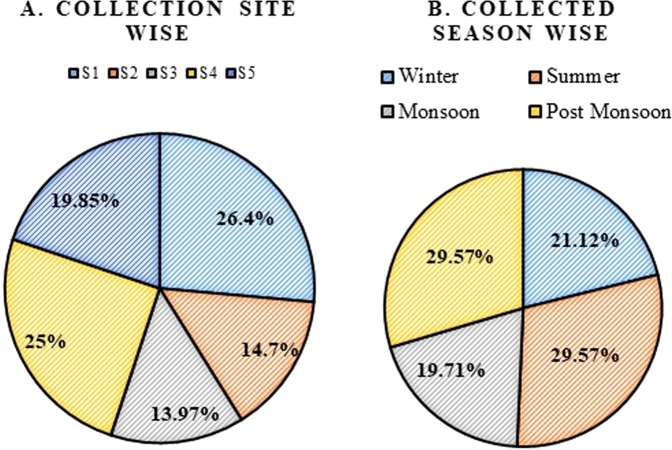
Percentage distribution of microalgae population. (**A**) Site wise. (**B**) Season Wise (winter, summer, monsoon, post-monsoon).

Abundance and diversity of microalgae species are influenced by physico - chemical parameters of that particular environment. Among the 142 cultures, Scenedesmaeae was found to be a dominant group with 52 species and members of Brachysiraceae were found low in number. The relative percentage of distribution of microalgae communities in our study was observed to be Scenedesmaceae (36.6%), Chlorellaceae (29.57%), Selenastraceae (9.85%), Asteromonadaceae (7.04%), Chlorococcaceae (4.92%), Brachysiraceae (0.70%), Bacillariaceae (3.52%), Sphaeropleaceae (1.4%), Oscillatoriaceae (2.81%) and Prasiolales incertae sedis (3.52%). Species distribution in these collection sites ranged from 13.97 to 26.4% with the maximum number of species recorded in site S1 (26.4%) followed by S4 (25%) (Fig. 1). Summer and post-monsoon season recorded a high percentage (29.57%) of microalgae population. Species composition was also found to be dominant in the post-monsoon season. The season-wise order of microalgae population dominance in the present study was observed as post-monsoon/summer > winter > monsoon.

**Figure 2:**
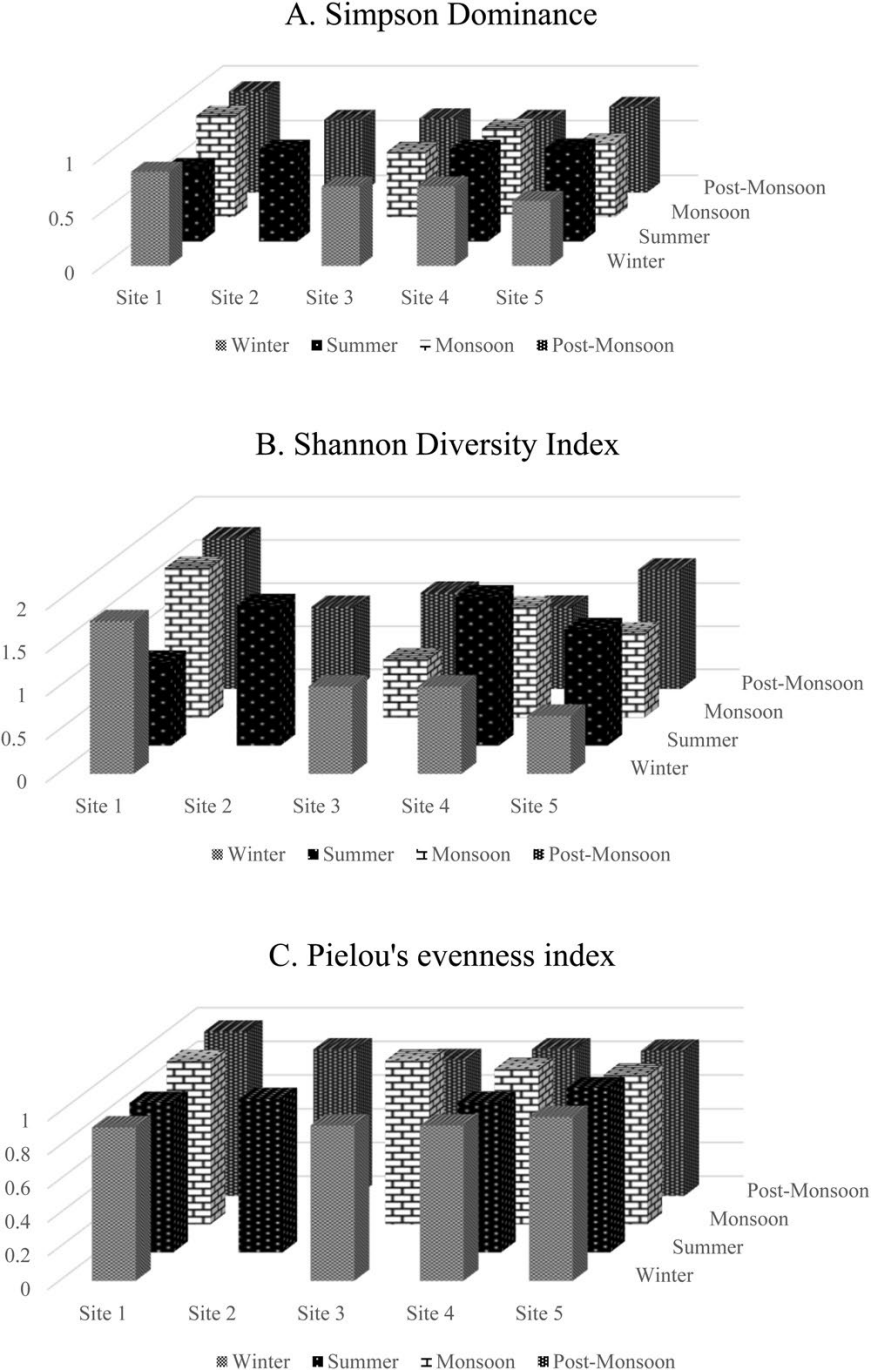
Microalgae diversity indices recorded during the different seasons in the study area. (**A**) Simpson Dominance index. (**B**) Shannon Diversity index. (**C**) Pielou’s evenness index.

Seasonal variations in diversity index and species evenness were illustrated in Fig. 2. The abundance of microalgae was found to be higher in summer and post-monsoon which was attributed to stable hydrographical parameters in the water. The Shannon diversity index H value for the collection sites was recorded in the range 0.673 to 1.77 with site S1 registered highest value (1.77) and site S5 with the least value (0.673) during the winter season (Fig. 2). The Simpson dominance index value was observed in the range of 0.6 to 0.928. Higher the dominance value lowers the diversity and vice versa. In our study, monsoon season was recorded with both high and low value of dominance in site S1 and S3 respectively which represented the low and high diversity of microalgae. Species evenness (0.80 to 0.975) and Margalef index (0.378 to 1.559) were observed during the study period representing the equitable distribution and abundance of microalgae species in the river Noyyal.

### Multivariate analysis

#### Correlation matrix analysis

**Figure 3:**
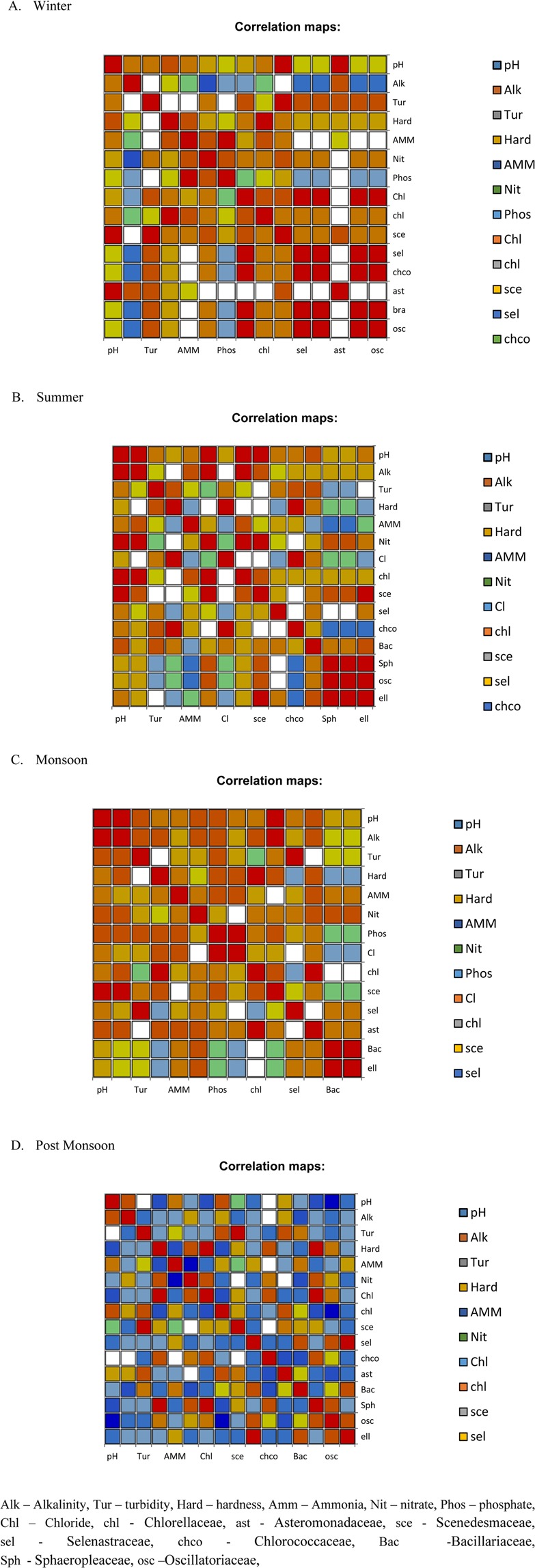
Summary of the results of Pearson correlation analysis. Correlation map between the physico-chemical parameters and microalgae population were represented in different colors. The red color indicates significant values. Correlation map for (**A**) Winter, (**B**) Summer, (**C**) Monsoon and (**D**) Post-monsoon season.

Pearson’s correlation coefficient was calculated between various physico-chemical parameters and the prevailing microalgae population to find their correlation during each season at river Noyyal (Table S1). During winter season, hardness parameter of water had shown positive correlation (increase) with Chlorellaceae (r = 0.947, p = 0.014). Also, Selenastraceae, Chlorococcaceae, Brachysiraceae and Oscillatoriaceae possessed a high positive correlation (increase) with chloride content (r = 0.968, p = 0.009) (Fig. 3A). During summer, pH of water showed positive correlation (increase) with alkalinity (r = 0.890, p = 0.043) and with Chlorellaceae (r = 0.897, p = 0.039). Alkalinity was found to possess high correlation with nitrate (r = 0.905, p = 0.035) and Chlorellaceae (r = 0.995, p = 0.000). Chlorellaceae and Scenedesmaceae were found to possess high positive correlation (increase) with nitrate content (r = 0.899, p = 0.038 and r = 0.933, p = 0.021) respectively (Fig. 3B). During monsoon season, pH showed high positive correlation with alkalinity (r = 0.942, p = 0.016) and phosphate with chloride (r = 0.903, p = 0.036). Also during this season, Scenedesmaceae showed high correlation with alkalinity (r = 0.964, p = 0.008) (Fig. 3C). During post-monsoon season, hardness showed high correlation with chloride (r = 1.0, p < 0.0001) and with Sphaeropleaceae (r = 1.0, p < 0.0001) (Fig. 3D).

### Principal component analysis

**Figure 4:**
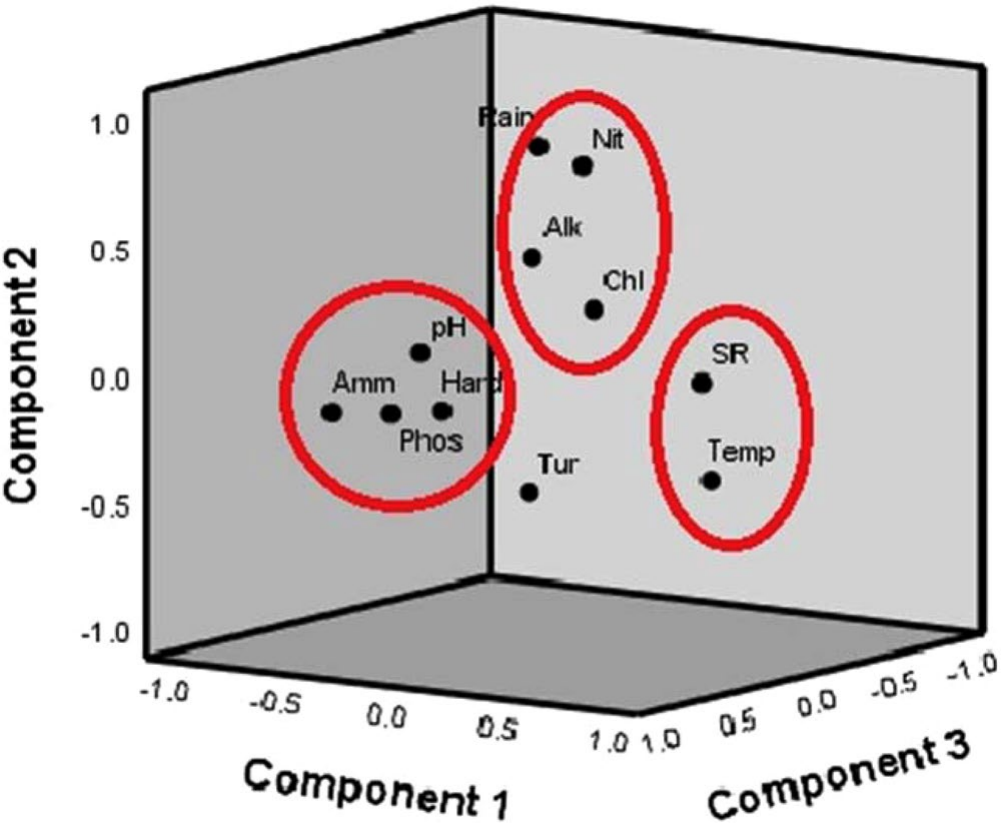
Principal Component Analysis for the Physico-chemical parameters during different seasons from the catchment region of river Noyyal. Nit – nitrate, Alk – Alkalinity, Chl – Chloride, SR – solar radiation, Temp – temperature, Tur – turbidity, Phos – phosphate, Hard – hardness, Amm – Ammonia.

The principal component analysis (PCA) identified four possible groups, which are accountable for the data structure, explaining 79.95% of the total variance of the dataset. This allowed to group the selected parameters according to the common features, as well as to access the incidence of each group on the overall variation in microalgae population (Fig. 4). PC1 explained 29.85% of the total variance with group 1 variables (alkalinity, nitrate, rain, and chlorides) showed significant positive correlation values. PC2 accounted for 20.46% variation with group 2 components (pH, hardness, ammonia, phosphates) showed a positive correlation with the second component and weak correlation values on the first component. PC3 included group 3 components (solar radiation and temperature) showed positive loading points with 16.31% of variation and PC4 form 13.32% variation with group 4 turbidity alone.

### Canonical Correspondence Analysis (CCA)

**Figure 5:**
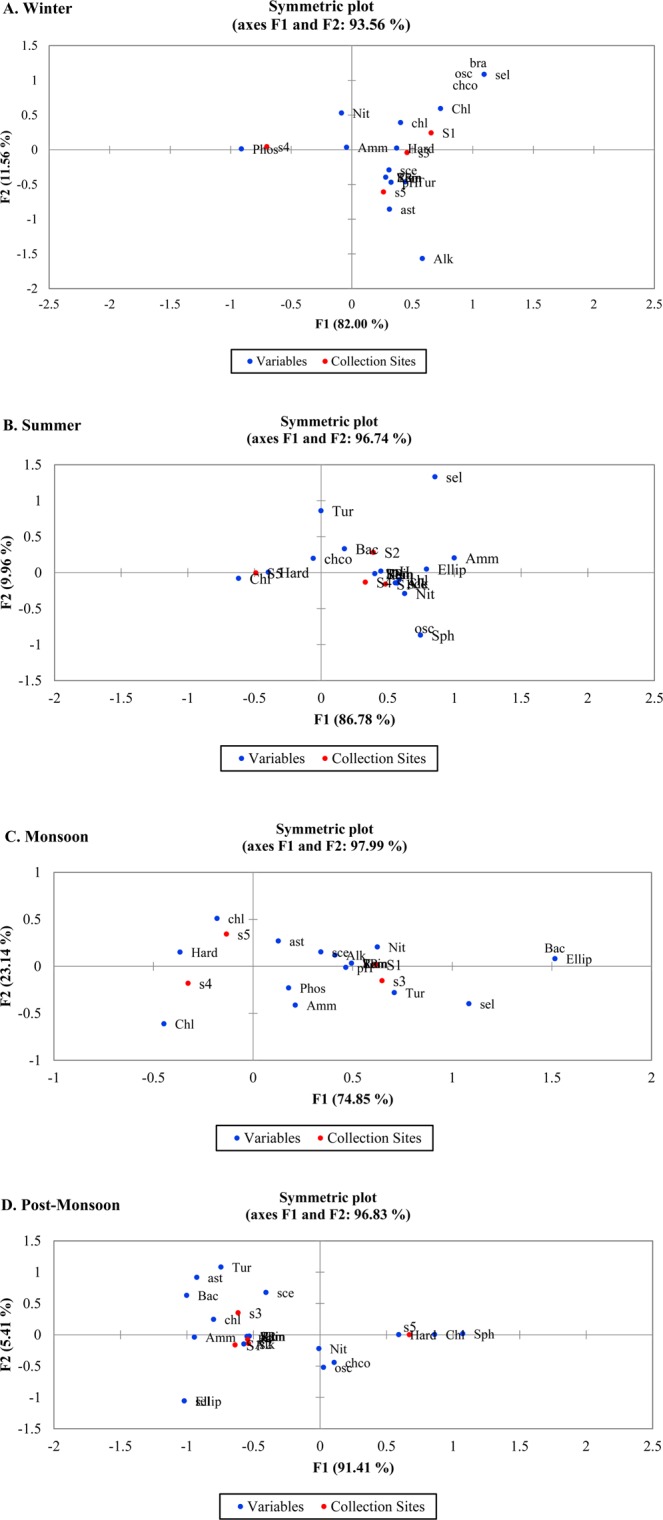
CCA plot showing the seasonal variation between the physic-chemical parameters and the microalgae population. (Red dot represents the collection sites and the blue dot represents the physico-chemical variables and microalgae cultures).

During the winter season, axis F1 and F2 explained 93.56% of the variability in the species-environment biplot (Fig. 5). Nitrate, phosphate, chloride, ammonia, and hardness had a positive correlation with site S1, S3, and S4. They were highly associated with Brachysiraceae, Oscillatoriaceae, Selenastraceae, Chlorococcaceae, and Chlorellaceae. The eigenvalues for the first two axes F1 and F2 were 0.359 and 0.051 respectively and inertia (%) values were observed as 81.99% and 11.55% respectively. Axis 1 displayed 82% of the variance in the species-environment relationship and was strongly influenced by pH, alkalinity, chloride, temperature, and rain. The second axis explained 11.56% of data set variability where ammonia and nitrate parameters were the strongest contributors to this axis. The analysis also revealed that Chlorellaceae, Brachysiraceae, Oscillatoriaceae, Selenastraceae, and Chlorococcaceae had the adaptability to hardness and require higher chloride and nitrate, lesser phosphate for their growth. Asteromonadaceae and Scenedesmaceae were adapted to higher alkalinity, temperature and rain, in addition, preferred lesser concentration of nitrate, phosphate, and ammonia.

In summer, 96.74% of the variance was observed from the axis F1 and F2 in CCA analysis. Among them, axis F1 contributed to 86.87% of variation with the eigenvalue of 0.197. pH, alkalinity, temperature, rain, solar radiation, nitrate, and ammonia were found to have a high correlation with site S1, S2, and S4 which were associated with Chlorellaceae, Scenedesmaceae, Selenastraceae, Chlorococcaceae, Bacillariaceae, Sphaeropleaceae and Oscillatoriaceae. Selenastraceae was influenced by high turbidity whereas Prasiolales incertae sedis, Chlorellaceae, Scenedesmaceae, Chlorococcaceae, and Bacillariaceae required nitrate and ammonia for their growth and also adapted to high rainfall, temperature, and solar radiation during summer. In the monsoon season, 97.99% of the variance was observed from axis F1 and F2, with F1, contributed to 74.85% of variation with the eigenvalue of 0.165. pH, alkalinity, turbidity, nitrate, phosphate, temperature, rain and solar radiation were found to have a high correlation with site S1 and S3. They were associated with Chlorellaceae, Scenedesmaceae, Selenastraceae, Bacillariaceae, Asteromonadaceae, and Prasiolales incertae sedis. The high concentration of nitrates influenced the growth of Scenedesmaceae and Astermonadaceae whereas the high concentration of phosphate and ammonia influenced the growth of Selenastraceae. Bacilliariaceae and Prasiolales incertae sedis were also influenced by F1 factors in monsoon season.

In post-monsoon season, 96.83% of the variance was observed from axis F1 and F2, with F1, contributed 91.41% of variation with the eigenvalue of 0.392. Hardness and chloride played a major factor in contributing to variation and found to have a high correlation with site S5 and associated with Chlorococcaceae, Sphaeropleaceae, and Oscillatoriaceae. The high concentration of nitrates influenced the growth of these cultures. Chlorellaceae, Asteromonadaceae, Scenedesmaceae, and Bacillariaceae were found to be predominant in site S3 and influenced by high turbidity and ammonia during post-monsoon season. Rain, temperature, solar radiation, pH and alkalinity were also found to possess equal contribution on site S1, S2, and S4.

## Discussion

Physico-chemical parameters are considered as one of the most important factors that are capable of influencing the aquatic environment. The diversity of microalgae distribution was mostly influenced by physico-chemical parameters of the water due to seasonal variations. Since there were only a few reports available on the assessment of physico-chemical parameters of Sirivani^11^ and no report on microalgae community of river Noyyal, our study serves as the baseline data on the prevailing conditions of microalgae and also emphasizes the influence of seasonal variation on microalgae population and distribution.

### Alkalinity of water promoting microalgae growth

pH regulates the acidic or basic characteristics of the aquatic ecosystem. All the biochemical functions and retention of physico-chemical properties of the water were prominently depended on the pH of the surrounding environments^12^. BIS^13^ has recommended an optimum level of pH as 7–8.5. In the present study, pH was found to be alkaline throughout the collection period except during winter for site S1 and S4. Higher pH observed in summer might have been attributed to the high photosynthetic activity of microalgae during that season. Pearson’s correlation coefficient showed pH of water possessed a positive correlation (increase) with alkalinity (r = 0.890, p = 0.043) and with Chlorellaceae (r = 0.897, p = 0.039) during summer (Fig. 3B). In the same season, alkalinity was also found to possess high correlation (increase) with nitrate (r = 0.905, p = 0.035) and Chlorellaceae (r = 0.995, p = 0.000). CCA analysis also revealed that Chlorellaceae, Scenedesmaceae, Selenastraceae, Chlorococcaceae, Bacillariaceae, Sphaeropleaceae, and Oscillatoriaceae were predominately found at site S1, S2, and S4 with high values of pH, alkalinity, temperature, rain, solar radiation, nitrate, and ammonia. Pearson’s correlation indicated hardness of water that had positive correlation (increase) with Chlorellaceae (r = 0.947, p = 0.014) and Sphaeropleaceae (r = 1.0, p < 0.0001) during monsoon (Fig. 3C) and post-monsoon (Fig. 3D) season respectively. These observations were in agreement with the findings of Sharma *et al*.^14^, Jindal and Sharma^15^.

### Nitrate as a key factor in prompting microalgae population

Nutrients such as phosphates, nitrates, nitrites, ammonia, and chloride in the freshwater exhibited considerable variation corresponding to the temporal climatic changes in each season which influenced the microalgae abundance and diversity. Nitrogen, a key variable for the growth of microalgae was provided in the form of ammonia, nitrite, and nitrate which was differently metabolized resulting in enhanced protein content and growth capacity of microalgae^16^. During post-monsoon season, nitrate N was found to be higher in all the collection sites which attributed to high rate of biological production, ammonia oxidation, terrestrial run-off^17^ and winter season registered lower concentration due to higher consumption of nitrate by the organisms in the freshwater^18^. CCA analysis also indicated that Chlorococcaceae, Sphaeropleaceae, and Oscillatoriaceae cultures showed a positive correlation (in terms of increase) at site S5 with a high concentration of nitrates during post-monsoon season. During winter season at site S1, S3 and S4 Brachysiraceae, Oscillatoriaceae, Selenastraceae, Chlorococcaceae, and Chlorellaceae showed a positive correlation (in terms of decrease) with a concentration of nitrate.

### Ammonia as an alternative N source for microalgae growth

The higher concentration of ammonia N at site S3 during all the seasons was mainly due to the intrusion of terrestrial runoff in the site. During summer, decreased concentration of ammonia N at S4 and S5 was observed which was attributed to rapid utilization of ammonia than nitrate by specific microalgae community at the given environment. It was assumed that the uptake of nitrate was inhibited in the presence of ammonium as the nitrate utilization requires energy for ammonium reduction. Moreover, microalgae prefer ammonia as a nitrogen source as the energetic costs of metabolic assimilation of nitrogen were less for nitrate. This was evident by the high correlation of ammonia with site S4 during the summer season in CCA. Chlorellaceae, Scenedesmaceae, Selenastraceae, Chlorococcaceae, Bacillariaceae, Sphaeropleaceae and Oscillatoriaceae prefer ammonia for its growth during summer. Generally, microalgae prefer ammonium for its growth as it requires less energy for assimilating into amino acids, but it is also reported that *Botryococcus braunii* and *Dunaliella tertiolecta* prefer nitrate over ammonium^19,20^. These results were in agreement with findings Dugdale^21^ and Thangaradjou *et al*.^22,23^.

### Influence of phosphate in microalgae growth

The high value of phosphates in the monsoon season was due to rain, surface water runoff, agricultural runoff and human activities which brought in a lot of inorganic phosphates. But, during summer and post-monsoon season, no phosphate content was observed which might be due to the buffering process of sediments under varying environmental conditions^24^. Algae have the capacity of controlling phosphate utilization, where it could be rapidly accumulated at the beginning of the growth phase and later slowly utilized in the subsequent life cycle. The process of algal phosphate uptake was influenced by environmental factors and physiological cell state such as light, temperature, pH, salinity, starvation, and growth^25^. CCA revealed that high concentration of phosphate influenced the growth of Selenastraceae, Bacilliariaceae and Prasiolales incertae sedis in monsoon season. Our findings also observed that phosphate content of stagnant water at sites S4 and S5 was found to be higher in the range of 54 mg/L and 854.34 mg/L, in particular, the highest value was observed in winter at site S4. Such higher values in the stagnant water during the winter season was in agreement with earlier findings^26,27^.

### Diversity indices in assessing species diversity

Microalgae species composition was observed to be dominant during the post-monsoon period (29.57%). This could be due to the terrestrial runoff during the monsoon season that might have brought in sufficient quantity of nutrients which successively increased the species composition^28^. Diversity of species was generally used to monitor ecological changes and expressed through indices. These indices were obtained and expressed as scores from quantitative data. There were many kinds of indices used in ecological studies, but the Shannon-Wiener Index (Hʹ) remains most frequently used as it was not significantly influenced by sample size and was helpful for indicating the pollution and trophic status of aquatic bodies^29,30^. The Hʹ value changes with ecological factors altering the diversity through changes in evenness but without changes in species richness^31^. In the present study, the Shannon diversity index H value recorded with moderate pollution at freshwater sites was in the range of 1–2 and high pollution for stagnant water with a range of 0 to 1. The relationship among the Hʹ index, the pollution level and diversity level as given by Shanthala *et al*.^32^, was concordant with our findings which have high diversity value in the collection sites with moderate pollution level.

High diversity value was found during the summer and post-monsoon season, which were considered as dry months and characterized by low water level due to evaporation which leads to the concentrated level of nutrients especially nitrates in these seasons. The reason might be the high diversity of microalgae population during this season. These findings were further supported by the low value of Simpson dominance index value and Margalef index value. Furthermore, lower diversity during monsoon season may have been attributed to the dilution of nutrient concentration. Our findings gained support from Miranda and Krishnakumar^33^ who reported the high Hʹ value during summer and low Hʹ value from the rainy season. Monsoon season recorded both high and low value of dominance in site S1 and S3 respectively which represented the low and high diversity of microalgae. These results were correlated with the findings of Miranda and Krishnakumar^33^ with higher dominance index value in monsoon season representing lower diversity. High species richness value (1.559) found during the post-monsoon season was due to a higher number of species recorded during this season. Chlorellaceae and Scenedesmaceae recorded higher Margalef index value during the study period. During monsoon season, owing to the variation in ecological and physico-chemical parameters, low species richness was observed. The results of our study correlated the previous findings^33–35^.

### PCA and CCA in determining the significant physico – chemical parameters

The usage of multivariate analysis was adequate to analyze the changes in microalgae communities and their associated physico-chemical parameters. PCA analysis of the physico-chemical parameters as statistical variables in this study demonstrated the influence of environmental and physico-chemical parameters in determining the quality of water. PC1 accounted for 29.85% of variance that had strong positive loadings on group1 variables and this might be due to the role of environmental variables influencing the water quality and their parameters. The findings of Zhou *et al*.^36,37^ were similar and agreeable with these results. The variance of 20.46% of PC2 variables included pH, chlorides, phosphates and hardness and these variations in the water were expected to be due to the involvement of anthropogenic activities. Results of CCA found that microalgae species in each season were closely associated with the environmental and physico-chemical parameters. In winter season, Brachysiraceae, Oscillatoriaceae, Selenastraceae, Chlorococcaceae, and Chlorellaceae have positive correlation with chemical parameters at site S1, S3 and S4. During summer, Chlorellaceae, Scenedesmaceae, Chlorococcaceae, and Bacillariaceae preferred nitrate and ammonia for their growth and also adapted to high rainfall, temperature, and solar radiation at site S1, S2 and S4. At site S1 and S3, Chlorellaceae, Scenedesmaceae, Selenastraceae, Bacillariaceae, Asteromonadaceae, and Prasiolales incertae sedis possess high correlation with pH, alkalinity, turbidity, nitrate, phosphate, temperature, rain and solar radiation in monsoon. Chlorellaceae, Asteromonadaceae, Scenedesmaceae, and Bacillariaceae were found to be predominant in site S3 and influenced by high turbidity and ammonia during post-monsoon season. Some species were found to tolerate and live at high-level variations in physico-chemical parameters whereas some cultures showed sensitivity to the minute changes in the parameters. Since different species differ in their environmental requirements, they responded differently to the variations in these variables. Our results were corroborated with Haridkar *et al*.^34^ findings, where they revealed that phytoplankton species distribution in Malvan Coast with seasonal shift was influenced by their significant fluctuation in physico-chemical parameters.

## Conclusion

Results from the study showed that there was a remarkable microalgae diversity in the catchment region of river Noyyal. This study summarized the seasonal fluctuations in physico-chemical parameters and microalgae diversity. pH, nitrate-nitrogen, and ammonia nitrogen were considered as favorable characteristics for the growth of microalgae. Scenedesmaceae (36.6%) and Chlorellaceae (29.57%) were found to be predominant during post-monsoon and summer seasons. Diversity indices revealed that microalgae were characterized by high Hʹ index, lower Simpson dominance value and Margalef index value with indefinite patterns of annual variations. Chlorellaceae and Scenedesmaceae were found equally distributed throughout all the seasons and revealed species evenness rather than species richness. PCA and CCA analysis revealed that pH, alkalinity, rain, nitrate, and ammonia nitrogen were the factors positively correlated with Chlorellaceae, Scenedesmaceae and Chlorococcaceae during summer and post-monsoon season. During winter and monsoon Brachysiraceae, Selenastraceae, Bacillariaceae and Chlorellaceae have high correlation with chemical parameters. Thus, the study provided a base data for the vibrant relationship between environmental parameters and microalgae population seasonally and concluded that catchment region of river Noyyal is more fertile with prodigious microalgae diversity.

## Materials and Methods

### Chemicals and reagents

All the chemicals and reagents used in the study were purchased from Hi-media laboratories, LLC, USA and glasswares procured from Borosil, India.

### Description of the study area and sample collection

**Figure 6:**
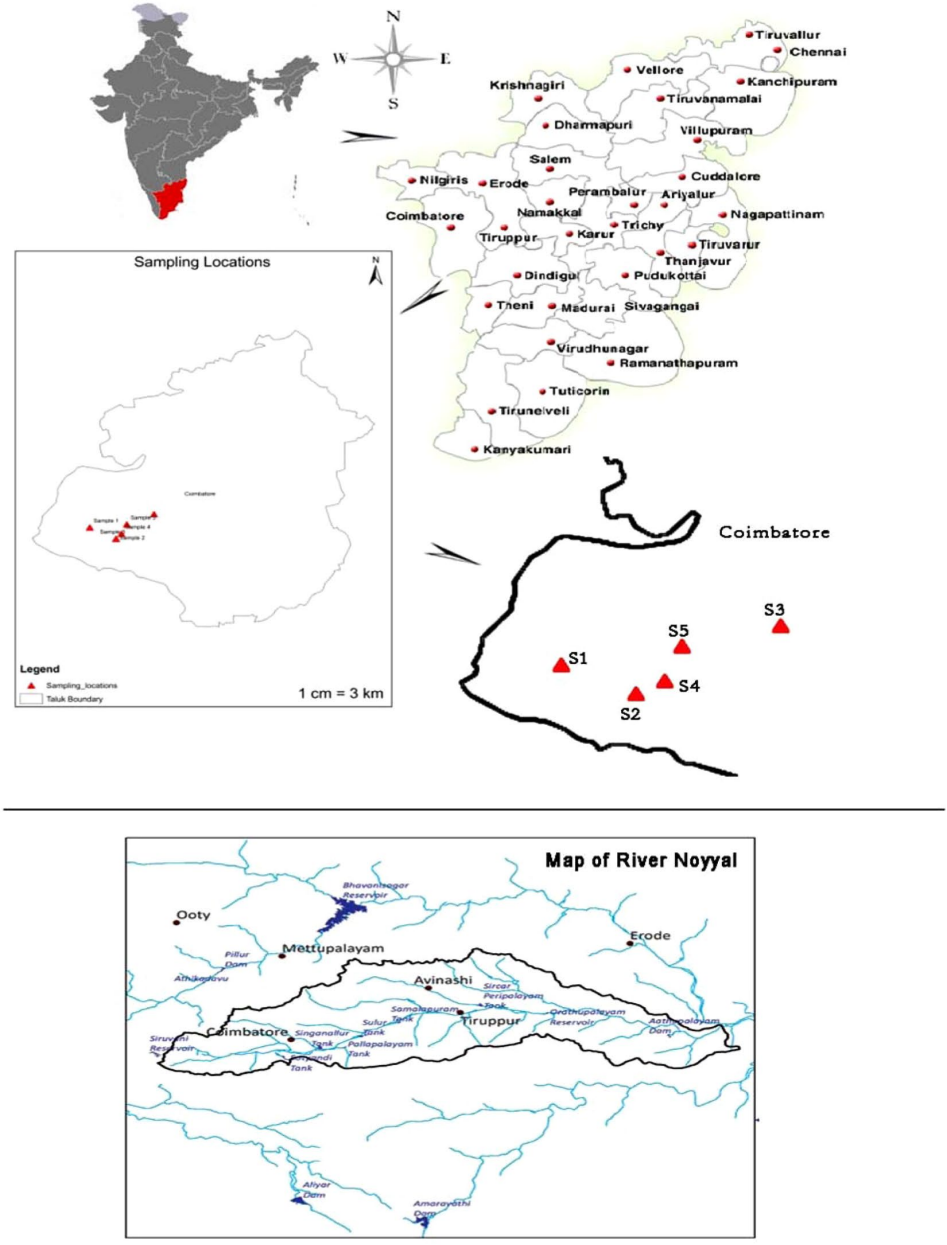
Map of river Noyyal showing the different sampling point of the study area. Note: S1 – Sadivayal Freshwater, S2 – Perumal kovilpathy freshwater, S3 – Irutupallam freshwater, S4 - Perumal kovilpathy stagnant water, S5 - Irutupallam stagnant water.

In the present investigation, three sampling sites from the catchment of river Noyyal at Siruvani Hills were chosen for microalgae isolation (Fig. 6). The Upstream site (Sadivayal check post S1– undisturbed site), midstream sites (Perumalkovilpathi freshwater S2 and Irutupallam freshwater S3- least disturbed sites) and downstream site (Perumalkovilpathi stagnant water S4 and Irutupallam stagnant water S5 – high disturbed site). The Distance between each collection site is approximately 5–10 Kms. The sampling sites lies in the following coordinates Sadivayal S1(10°94′39.5″N and 76°72′15″ E), Perumalkovilpathy S2 (10°92′75.8″ N and 76°75′51″ E), Iruttupallam S3 (10°94′85.1″ N 76°76′92.8″ E), Perumalkovilpathy stagnant water S4 (10°92′78.5″ N and 76°75′54.5″ E) and Irutupallam stagnant water S5 (10°96′34.8″ N and 76°80′43.6″ E). The samples were collected corresponding to four different seasons of South India viz., in the month of January, April, July and October 2014 implying winter, summer, monsoon and post-monsoon seasons. Water samples were collected at different depths and passed through an upward series of plankton nets of mesh sizes 10, 30 and 120 µm to collect microalgal species from the water sample.

### Physico-chemical analysis of water samples

The water sample was collected, transported and analyzed for physico-chemical parameters according to standard APHA protocol^38^. All analysis were ensured with known standards and experiments done in triplicates. The pH and electrical conductivity (EC) were measured using digital pH meter (Hanna, India). The turbidity of water was measured nephelometrically and expressed in NTU. The titrimetric method was used to analyze hardness (EDTA titration), alkalinity (phenolphthalein and methyl orange titration) and chloride (Mohr’s method). Phosphate, nitrate-nitrogen, and ammonia nitrogen were analyzed using UV-VIS spectrophotometer (Hitachi, U 2910-2J1-0012, Japan). Phosphate was analyzed by stannous chloride method, nitrate was measured using brucine – sulphanilic acid method and ammonia nitrogen by Nessler’s method following standard procedures.

### Microalgae isolation and pure culture maintenance

Microalgae were isolated from water samples using traditional isolation techniques like single-cell isolation by capillary pipette, serial dilution, culture enrichment method, pour plate and spread plate methods. Different growth media was used for the isolation viz. Bold Basal, CFTRI, Euglena, Fogg’s, modified Fogg’s media with 0.2% KNO_3_, Allen, BG-11 and CHU media to isolate the possible number of cultures. The composition and concentration of the media were referred from UTEX and given in Supplementary Table (S2). Single-cell isolation technique was performed with 10 µl of water samples placed on the cavity slide and observed using a light microscope (Accuscope, USA) under 10X and 40X magnification. The specific cell was picked up using a thin flamed capillary tube inserted into 10 µl microtip and inoculated in the growth media. Serial dilution was performed with water samples serially diluted to 10°, 10^−1^,10^−2^, 10^−3^, 10^−4^ dilutions and inoculated in growth media. Enrichment method was performed by adding an equal volume of water sample to the sterile growth media and incubated in the culture room. All the cultures were maintained by frequent subculture in the algae culture room at 28 ± 2 °C under 27 to 37 µmol m^−2^ s^−1^ with a light and dark cycle of 16:8 hours.

In order to obtain a pure culture, repeated dilution was followed. Each dilution was subsequently subjected to four-phase streaking on an agar plate and incubated in the culture room (as mentioned above) until colonies have appeared and the growth was monitored using a microscope for every 24 hr. Re-streaking or repeated dilution was performed to avoid any mix of cultures. Pure cultures obtained were coded as Karunya Algae Culture Collection (KACC) and maintained in their respective medium in the culture room.

### Diversity Index

Diversity indices were computed using Microsoft Excel 2013 to find out the diversity of microalgae assemblages during the collection period. Shannon-Wiener diversity index (H′), Simpson dominance, Margalef’s index and Pielou evenness (J′) were calculated as follows.

### Shannon–Weiner index

Species diversity was calculated using Shannon–Weiner diversity index, Hʹ^39^:

$$H=-\,\mathop{\sum }\limits_{i=1}^{S}{p}_{i}\,\mathrm{ln}({p}_{i})$$

where ‘p_i_’ is the relative abundance of species ‘i’, calculated as the proportion of individuals of a given species to the total number of individuals in the community, and ‘S’ is the total number of species:

$${p}_{i}={n}_{i}/N$$

where ‘n_i_’ is the number of individuals of species i, and ‘N’ represents the total number of individuals of all species.

### Simpson’s dominance index

Species dominance was calculated using Simpson’s dominance index^40^:

$$D=\mathop{\sum }\limits_{i=1}^{S}{p}_{i}^{2}$$

where p_i_ is the relative abundance of species i and S is the total number of species.

### Margalef’s index

Specific diversity was calculated using Margalef’s index^41^ which is expressed as

$$D=(S-1)/\,\mathrm{ln}\,N$$

where S is the species number and N is the total number of individuals.

### Pielou’s evenness index

Species evenness (*J*) was calculated using the Pielou’s evenness index^42^:

$$J=\frac{H}{Hmax}$$

where ‘H’ is the number derived from the Shannon diversity index and ‘Hmax’ is the maximum value of H.

### Statistical analysis

Different statistical analyses such as Principal Component Analysis (PCA), Canonical correspondence analysis (CCA) and Pearson’s correlation matrix (r) were applied to analyze the diversity of microalgae population and to find out the differences between the sites. Multivariate analyses were used (1) to identify environmental parameters that were most strongly associated with each other and (2) to define environmental factors to phytoplankton species associations. Principal component analysis (PCA)^33^ was computed using IBM SPSS to identify trends between highly correlating physico - chemical parameters. CCA was computed using XLSTAT 2018 for Windows to examine the environmental variables influencing the microalgae community. Pearson’s correlations were carried out using XLSTAT 2018 to assess the correlation between the environmental variables and microalgae communities.

## Supplementary information

Supplementary table S1 and S2

## Data Availability

The datasets generated during and/or analyzed during the current study are available from the corresponding author on reasonable request.
