# Supplementary material for: Seasonal distribution and population dynamics of limnic microalgae and their association with physico-chemical parameters of river Noyyal through multivariate statistical analysis
Source: Sci Rep. 2019 Oct 21;9:15021. doi: 10.1038/s41598-019-51542-w (PMC6803665; doi:10.1038/s41598-019-51542-w)
Supplement: Supplementary file 1 — Supplementary table S1 and S2 [file 41598_2019_51542_MOESM1_ESM.pdf]

Seasonal distribution and population dynamics of limnic microalgae and their association with physico-chemical parameters of river Noyyal through multivariate statistical analysis

Pandian SureshKumar and Jibu Thomas*

Algae Biomass Research Laboratory, Department of Biotechnology, Karunya Institute of Technology and Sciences, Coimbatore 641114 Tamil Nadu, India

**Email – jibuthomas.t@gmail.com*

Table S1. Summary of the results of Pearson correlation analysis. Correlation values between the physico-chemical parameters and microalgae population were given. Significant values were marked in **BOLD**. A. Winter, B. Summer, C. Monsoon and D. Post-monsoon season. Alk – Alkalinity, Tur – turbidity, Hard – hardness, Amm – Ammonia, Nit – nitrate, Phos – phosphate, Cl – Chloride, chl - Chlorellaceae, ast - Asteromonadaceae, sce - Scenedesmaceae, sel - Selenastraceae, chco - Chlorococcaceae, brac - Brachysiraceae, Bac -Bacillariaceae, Sph - Sphaeropleaceae, osc –Oscillatoriaceae, pi - Prasiolales incertae sedis

A. Winter

Variables	pH	Alk	Tur	Hard	AMM	Nit	Phos	Cl	chl	sce	sel	chco	ast	brac	osc
pH	**1**	0.552	0.520	0.725	0.550	0.264	0.171	0.367	0.538	0.826	0.192	0.192	0.837	0.192	0.192
Alk	0.552	**1**	0.028	0.151	-0.147	-0.637	-0.328	-0.324	-0.151	0.090	-0.405	-0.405	0.705	-0.405	-0.405
Tur	0.520	0.028	**1**	0.085	-0.001	0.503	-0.065	0.607	0.108	0.847	0.678	0.678	0.640	0.678	0.678
Hard	0.725	0.151	0.085	**1**	0.727	0.349	0.171	0.566	**0.947**	0.553	0.319	0.319	0.232	0.319	0.319
AMM	0.550	-0.147	-0.001	0.727	**1**	0.656	0.801	0.211	0.693	0.494	-0.006	-0.006	0.167	-0.006	-0.006
Nit	0.264	-0.637	0.503	0.349	0.656	**1**	0.635	0.590	0.509	0.666	0.553	0.553	0.036	0.553	0.553
Phos	0.171	-0.328	-0.065	0.171	0.801	0.635	**1**	-0.193	0.164	0.238	-0.290	-0.290	0.056	-0.290	-0.290
Cl	0.367	-0.324	0.607	0.566	0.211	0.590	-0.193	**1**	0.726	0.667	**0.962**	**0.962**	0.054	**0.962**	**0.962**
chl	0.538	-0.151	0.108	**0.947**	0.693	0.509	0.164	0.726	**1**	0.522	0.516	0.516	0.000	0.516	0.516
sce	0.826	0.090	0.847	0.553	0.494	0.666	0.238	0.667	0.522	**1**	0.590	0.590	0.699	0.590	0.590
sel	0.192	-0.405	0.678	0.319	-0.006	0.553	-0.290	**0.962**	0.516	0.590	**1**	**1.000**	0.000	**1.000**	**1.000**
chco	0.192	-0.405	0.678	0.319	-0.006	0.553	-0.290	**0.962**	0.516	0.590	**1.000**	**1**	0.000	**1.000**	**1.000**
ast	0.837	0.705	0.640	0.232	0.167	0.036	0.056	0.054	0.000	0.699	0.000	0.000	**1**	0.000	0.000
brac	0.192	-0.405	0.678	0.319	-0.006	0.553	-0.290	**0.962**	0.516	0.590	**1.000**	**1.000**	0.000	**1**	**1.000**
osc	0.192	-0.405	0.678	0.319	-0.006	0.553	-0.290	**0.962**	0.516	0.590	**1.000**	**1.000**	0.000	**1.000**	**1**

Values in bold are different from 0 with a significance level alpha=0.05

B. Summer

Variables	pH	Alk	Tur	Hard	AMM	Nit	Cl	chl	sce	sel	chco	Bac	Sph	osc	pi
pH	**1**	**0.890**	0.435	0.328	0.423	0.807	0.230	**0.897**	0.854	0.438	0.426	0.641	0.316	0.316	0.488
Alk	**0.890**	**1**	0.104	0.088	0.659	**0.905**	0.028	**0.995**	0.789	0.197	0.313	0.225	0.223	0.223	0.291
Tur	0.435	0.104	**1**	0.620	0.117	-0.180	0.557	0.126	0.000	0.581	0.753	0.671	-0.399	-0.399	-0.068
Hard	0.328	0.088	0.620	**1**	-0.340	-0.062	**0.992**	0.029	0.024	-0.203	0.823	0.561	-0.123	-0.123	-0.206
AMM	0.423	0.659	0.117	-0.340	**1**	0.397	-0.357	0.693	0.198	0.370	0.215	-0.281	-0.401	-0.401	-0.172
Nit	0.807	**0.905**	-0.180	-0.062	0.397	**1**	-0.128	**0.899**	**0.933**	0.111	-0.036	0.257	0.603	0.603	0.581
Cl	0.230	0.028	0.557	**0.992**	-0.357	-0.128	**1**	-0.038	-0.073	-0.308	0.820	0.454	-0.185	-0.185	-0.311
Chl	**0.897**	**0.995**	0.126	0.029	0.693	**0.899**	-0.038	**1**	0.799	0.286	0.276	0.241	0.221	0.221	0.332
Sce	0.854	0.789	0.000	0.024	0.198	**0.933**	-0.073	0.799	**1**	0.318	-0.066	0.553	0.739	0.739	0.800
Sel	0.438	0.197	0.581	-0.203	0.370	0.111	-0.308	0.286	0.318	**1**	0.000	0.527	0.000	0.000	0.484
Chco	0.426	0.313	0.753	0.823	0.215	-0.036	0.820	0.276	-0.066	0.000	**1**	0.327	-0.535	-0.535	-0.468
Bac	0.641	0.225	0.671	0.561	-0.281	0.257	0.454	0.241	0.553	0.527	0.327	**1**	0.408	0.408	0.612
Sph	0.316	0.223	-0.399	-0.123	-0.401	0.603	-0.185	0.221	0.739	0.000	-0.535	0.408	**1**	**1.000**	0.875
Osc	0.316	0.223	-0.399	-0.123	-0.401	0.603	-0.185	0.221	0.739	0.000	-0.535	0.408	**1.000**	**1**	0.875
Pi	0.488	0.291	-0.068	-0.206	-0.172	0.581	-0.311	0.332	0.800	0.484	-0.468	0.612	0.875	0.875	**1**

Values in bold are different from 0 with a significance level alpha=0.05

C. Monsoon

Variables	pH	Alk	Tur	Hard	AMM	Nit	Phos	Cl	chl	sce	sel	ast	Bac	Pi
pH	**1**	**0.942**	0.783	0.540	0.507	0.712	0.784	0.453	0.503	0.853	0.574	0.629	0.234	0.234
Alk	**0.942**	**1**	0.609	0.605	0.281	0.714	0.650	0.300	0.683	**0.964**	0.338	0.663	0.110	0.110
Tur	0.783	0.609	**1**	0.096	0.320	0.340	0.648	0.345	-0.110	0.542	0.848	0.048	0.117	0.117
Hard	0.540	0.605	0.096	**1**	0.448	0.182	0.767	0.764	0.859	0.658	-0.297	0.707	-0.329	-0.329
AMM	0.507	0.281	0.320	0.448	**1**	0.439	0.692	0.725	0.250	0.096	0.395	0.658	0.480	0.480
Nit	0.712	0.714	0.340	0.182	0.439	**1**	0.244	-0.061	0.470	0.525	0.475	0.755	0.753	0.753
Phos	0.784	0.650	0.648	0.767	0.692	0.244	**1**	**0.903**	0.445	0.621	0.326	0.520	-0.123	-0.123
Cl	0.453	0.300	0.345	0.764	0.725	-0.061	**0.903**	**1**	0.355	0.297	0.060	0.416	-0.251	-0.251
chl	0.503	0.683	-0.110	0.859	0.250	0.470	0.445	0.355	**1**	0.708	-0.383	0.840	-0.086	-0.086
sce	0.853	**0.964**	0.542	0.658	0.096	0.525	0.621	0.297	0.708	**1**	0.165	0.542	-0.147	-0.147
sel	0.574	0.338	0.848	-0.297	0.395	0.475	0.326	0.060	-0.383	0.165	**1**	0.000	0.559	0.559
ast	0.629	0.663	0.048	0.707	0.658	0.755	0.520	0.416	0.840	0.542	0.000	**1**	0.408	0.408
Bac	0.234	0.110	0.117	-0.329	0.480	0.753	-0.123	-0.251	-0.086	-0.147	0.559	0.408	**1**	**1.000**
pi	0.234	0.110	0.117	-0.329	0.480	0.753	-0.123	-0.251	-0.086	-0.147	0.559	0.408	**1.000**	**1**

Values in bold are different from 0 with a significance level alpha=0.05

D. Post-Monsoon

Variables	pH	Alk	Tur	Hard	AMM	Nit	Chl	cl	sce	sel	chco	ast	Bac	Sph	osc	ell
pH	**1**	0.675	0.016	-0.653	0.421	-0.265	-0.652	0.773	-0.191	-0.408	0.045	0.288	-0.318	-0.668	**-0.879**	-0.408
Alk	0.675	**1**	-0.409	-0.278	-0.274	0.371	-0.292	0.300	-0.507	-0.344	0.013	0.268	-0.689	-0.305	-0.530	-0.344
Tur	0.016	-0.409	**1**	-0.256	0.118	-0.392	-0.259	0.632	0.833	-0.286	-0.491	0.724	0.572	-0.254	-0.441	-0.286
Hard	-0.653	-0.278	-0.256	**1**	-0.610	0.764	**1.000**	-0.738	0.279	-0.272	0.614	-0.391	-0.431	**1.000**	0.594	-0.272
AMM	0.421	-0.274	0.118	-0.610	**1**	**-0.886**	-0.590	0.380	-0.114	0.314	0.025	-0.282	0.458	-0.592	-0.227	0.314
Nit	-0.265	0.371	-0.392	0.764	**-0.886**	**1**	0.750	-0.479	0.012	-0.476	0.423	-0.035	-0.779	0.745	0.220	-0.476
Chl	-0.652	-0.292	-0.259	**1.000**	-0.590	0.750	**1**	-0.740	0.279	-0.266	0.627	-0.409	-0.425	**1.000**	0.599	-0.266
cl	0.773	0.300	0.632	-0.738	0.380	-0.479	-0.740	**1**	0.304	-0.408	-0.389	0.722	0.167	-0.748	**-0.944**	-0.408
sce	-0.191	-0.507	0.833	0.279	-0.114	0.012	0.279	0.304	**1**	-0.559	0.000	0.456	0.228	0.280	-0.228	-0.559
sel	-0.408	-0.344	-0.286	-0.272	0.314	-0.476	-0.266	-0.408	-0.559	**1**	-0.408	-0.408	0.612	-0.250	0.612	**1.000**
chco	0.045	0.013	-0.491	0.614	0.025	0.423	0.627	-0.389	0.000	-0.408	**1**	-0.667	-0.667	0.612	0.167	-0.408
ast	0.288	0.268	0.724	-0.391	-0.282	-0.035	-0.409	0.722	0.456	-0.408	-0.667	**1**	0.167	-0.408	-0.667	-0.408
Bac	-0.318	-0.689	0.572	-0.431	0.458	-0.779	-0.425	0.167	0.228	0.612	-0.667	0.167	**1**	-0.408	0.167	0.612
Sph	-0.668	-0.305	-0.254	**1.000**	-0.592	0.745	**1.000**	-0.748	0.280	-0.250	0.612	-0.408	-0.408	**1**	0.612	-0.250
osc	**-0.879**	-0.530	-0.441	0.594	-0.227	0.220	0.599	**-0.944**	-0.228	0.612	0.167	-0.667	0.167	0.612	**1**	0.612
ell	-0.408	-0.344	-0.286	-0.272	0.314	-0.476	-0.266	-0.408	-0.559	**1.000**	-0.408	-0.408	0.612	-0.250	0.612	**1**

Values in bold are different from 0 with a significance level alpha=0.05

Table S2 Composition of culture growth media

CFTRI Media (pH: 10.0)

**Constituents**	**g/L**
$NaHCO_3$	4.5
K_2HPO_4	0.5
$NaNO_3$	1.5
NaCl	1.0
$MgSO_4.7H_2O$	0.2
$CaCl_2$	0.04
$FeSO_4$	0.018
K_2SO_4	1.0

Fogg's Medium (pH: 7.5)

**Constituents**	**g/L**
$MgSO_4.7H_2O$	0.2
K_2HPO_4	0.2
Micro Nutrients	1 ml
$CaCl_2$	0.1
Fe- EDTA solution	5.0 ml
**Micro Nutrients**	
H_3BO_3	0.286
$MnCl_2.4H_2O$	0.181
$ZnSO_4.7H_2O$	0.022
$CuSO_4.5H_2O$	0.008
$Na_2MoO_4.2\ H_2O$	0.039

Modified Fogg Medium – Addition of 0.2% KNO_3 to the Fogg's medium

Bold Basal (BB) MEDIA (pH 6.6)

**Constituents**	**Solutions**	**g/L**
$NaNO_3$	Solution 1	250 mg
$CaCl_2$. $2H_2O$	Solution 2	25 mg
$MgSO_4.7H_2O$	Solution 2	75 mg
NaCl	Solution 2	25 mg
KH_2PO_4	Solution 3	175 mg
K_2HPO_4	Solution 3	75 mg
EDTA	Solution 4	50 mg
KOH	Solution 4	31.mg
$FeSO_4.7H_2O$	Solution 5	5 mg
H_2SO_4	Solution 5	1 µl
H_3BO_3	Solution 6	11 mg
Micronutrients		
$ZnSO_4.7H_2O$	Solution 7	8.82g/L
$MnCl_2.4H_2O$	Solution 7	1.44g/L
MoO_3	Solution 7	11.42
$CuSO_4$. H_2O	Solution 7	1.57
$Ca(NO_3)_2.6H_2O$	Solution 7	0.49

Commercial Media

Allen Basal solution (Cat. No. PL037),

BG- 11 Basal Solution (Cat. No. PL030) and

CHU basal solution (Cat. No. PL032) were purchased from HIMEDIA.
